# Séroprévalence des anticorps anti-SARS-CoV-2 parmi les voyageurs et travailleurs dépistés à la clinique Saint Luc de Bukavu, à l´Est de la République Démocratique du Congo, de mai en août 2020

**DOI:** 10.11604/pamj.2021.38.93.26663

**Published:** 2021-01-27

**Authors:** Philippe Bianga Katchunga, Aimé Murhula, Prince Akilimali, Jean Claude Zaluka, Racine Karhikalembu, Mack Makombo, Justin Bisimwa, Eugene Mubalama

**Affiliations:** 1 Clinique Saint-Luc de Bukavu, Bukavu, République Démocratique du Congo,; 2 Faculté de Médecine, Université Officielle de Bukavu, Bukavu, République Démocratique du Congo

**Keywords:** Séroprévalence, SARS-CoV-2, Bukavu, Congo, Seroprevalence, SARS-CoV-2, Bukavu, Congo

## Abstract

**Introduction:**

les tests sérologiques anti-SARS-CoV-2 pourrait jouer un rôle majeur dans l´estimation de la prévalence de la COVID-19. L´objectif était d´estimer la prévalence de la COVID-19 dans la ville de Bukavu, à l'Est de la République Démocratique du Congo, parmi les voyageurs et travailleurs.

**Méthodes:**

entre mai et août 2020, les tests rapides Cellex qSARS-CoV-2 IgG/IgM (Cellex, Inc., USA), test immunologique à flux latéral, ont été utilisés pour détecter et différencier des anticorps anti-SARS-CoV-2 chez les voyageurs et les travailleurs en quête d´un certificat médical.

**Résultats:**

parmi 684 habitants de la ville de Bukavu dépistés de la COVID-19 (4,2% hispaniques, 2,8% autres africains, 0,9% asiatiques), la séroprévalence anti-SARS-CoV-2 était de 40,8% (IgG+/IgM+: 34,6%; IgG+/IgM-: 0,5%; IgG-/IgM+: 5,4%). La séroprévalence cumulée des IgG anti-SARS-CoV-2 est passée de 24,5% à 35,2% de mai à août 2020. Les prédicteurs indépendants des anticorps anti-SARS-CoV-2 étaient l´âge > 60 ans [OR ajusté= 2,07(1,26-3,38)] et la non-appartenance au personnel médical [OR ajusté= 2,28(1,22-4,26)]. Treize virgule neuf pour cent (13,9%) des séropositifs pour les SARS-CoV-2 étaient symptomatiques et hospitalisés.

**Conclusion:**

la présente étude montre une séroprévalence très élevée des anticorps anti-SARS-CoV-2 dans la ville de Bukavu, à l´Est de la République Démocratique du Congo, parmi les voyageurs et travailleurs, pouvant impacter positivement sur l´immunité communautaire de la population étudiée. Ainsi, la prise en charge de la COVID-19 devrait être contextualisée en fonction des réalités de chaque région.

## Abstract (en)

**Introduction:**

SARS-CoV-2 serology tests could play a crucial role in estimating the prevalence of COVID-19. The purpose of this study was to estimate the prevalence of COVID-19 among travellers and workers in Bukavu, a city in eastern Democratic Republic of the Congo.

**Methods:**

between May and August 2020, the Cellex qSARS-CoV-2 IgG/IgM Rapid Test (Cellex, Inc., USA), lateral flow immunoassay was used to rapidly detect and differentiate antibodies against SARS-CoV-2 among travellers and workers seeking medical certification.

**Results:**

among the 684 residents of the city of Bukavu screened for COVID-19 (4.2% Hispanic, 2.8% other African, 0.9% Asian), the seroprevalence anti-SARS-CoV-2 antibodies was 40.8% (IgG+/IgM+: 34.6%; IgG+/IgM-: 0.5%; IgG-/IgM+: 5.4%). Cumulative seroprevalence of anti-SARS-CoV-2 IgG antibodies increased from 24.5% to 35.2% from May to August 2020. Independent predictors of SARS-CoV-2 antibodies were age > 60 years [adjusted OR = 2.07(1.26-3.38)] and non-membership of the medical staff [adjusted OR = 2.28 (1.22-4.26)]. Thirteen point nine percent of patients seropositive for SARS-CoV-2 antibodies were symptomatic and hospitalized.

**Conclusion:**

this study shows a very high seroprevalence of SARS-CoV-2 antibodies among travellers and workers in Bukavu, a city in eastern Democratic Republic of the Congo, which may positively affect community immunity in the study population. Thus, the management of COVID-19 should be contextualized according to local realities.

## Introduction

La maladie à coronavirus 2019 (COVID-19) est actuellement un problème de santé publique mondial majeur. En effet, avec un indice de contagion de base (R0) variant entre 2 et 3.5 [1], le syndrome respiratoire aigu sévère lié au coronavirus 2 (SARS-CoV-2) s´est répandu très rapidement dans le monde entier. Ainsi, peu après son apparition en décembre 2019 à Wuhan en Chine [2], l'épidémie due au SARS-CoV-2 a été déclarée pandémie le 11 mars 2020 par l'organisation mondiale de la santé (OMS) [3]. A la date du 09 octobre 2020, un cumul de 37 millions de cas a été enregistré parmi lesquels 1 million des décès [4].

Le diagnostic des personnes exposées ou infectées par le SARS-CoV-2 est essentiel pour contrôler la pandémie mondiale de COVID-19. Dans une ville où la maladie s´est répandue, il est important d´estimer la prévalence de la maladie en vue d´évaluer l´immunité collective.

Actuellement, la détection de l'ARN viral par RT-PCR est la méthode validée pour confirmer le diagnostic d'infection par le SARS-CoV-2 dans la pratique. D´autre part, les tests sérologiques pour détecter la présence d´anticorps anti-SARS-CoV-2 visent à identifier une infection antérieure par le SARS-CoV-2 et peuvent aider à confirmer la présence d´une infection récente en cas de limite de la PCR [5, 6].

La région d´Afrique subsaharienne (ASS) enregistre un nombre faible des cas de la COVID-19 et surtout une mortalité très faible comparée à d´autres régions du monde [4]. Deux raisons majeures pourraient expliquer cette observation: une population jeune et une immunité croisée possible entre le SARS-CoV-2 et d´autres agents infectieux très fréquents dans la région. En raison d'un niveau socio-économique faible de sa population, les mesures de prévention contre la COVID-19 notamment la distanciation sociale et le port obligatoire de masque n'ont jamais été respectées [7]. Il est donc possible que le taux de contamination asymptomatique par le SARS-CoV-2 soit très élevé mais non documenté du fait de la limite de dépistage par le RT-PCR. Ainsi, le présent travail s'est assigné comme objectif d'estimer la prévalence de la COVID-19 en analysant les résultats des tests sérologiques réalisés dans la ville de Bukavu, à l'Est de la République Démocratique du Congo (RDC), parmi les voyageurs et travailleurs dépistés à la clinique Saint Luc de Bukavu.

## Méthodes

**Population étudiée:** la présente étude transversale s'est déroulée à la clinique Saint Luc de Bukavu (CSL/B), un centre du niveau tertiaire, entre le 15 mai 2020 et le 30 août 2020. La CSL/B est l'un des 3 centres de dépistage et prise en charge de la COVID-19 dans la ville de Bukavu. Tout sujet, quel que soit l´âge, le sexe et l´origine était éligible pour un dépistage sérologique volontaire contre le SARS-CoV-2. Le motif de dépistage était l´obtention d´un certificat de voyage et la connaissance de son statut immunitaire pour des raisons professionnelles ou une mise au point clinique chez des sujets symptomatiques.

**Collecte des données:** le sujet asymptomatique était envoyé au laboratoire pour le test sérologique après contrôle de la température et saturation en oxygène. En cas des IgM+/IgG-, un prélèvement nasal pour le RT-PCR était effectué et le patient était isolé à domicile et traité. Le sujet symptomatique était orienté vers la clinique de la Fièvre de la CSL/B où un médecin procédait à la collecte des données cliniques (symptômes, température, saturation en oxygène et examen physique). Une prise de sang pour des analyses biologiques était également réalisée.

**Analyse biologique:** une ponction veineuse de 3 ml a été effectuée chez le candidat au dépistage. Après centrifugation, le sérum a été utilisé pour la détection qualitative et différenciation des anticorps IgM et IgG anti-SARS-CoV-2 par le test rapide Cellex qSARS-CoV-2 IgG/IgM (Cellex, Inc., USA), un test immunologique à flux latéral. La sensibilité, la spécificité, la valeur prédictive positive et la valeur prédictive négative (pour une prévalence de 5%) sont respectivement de 93,8%, 96,0%, 55,2% et 99,7%. Aucune réaction croisée n´est observée avec ce test. Celui-ci est approuvé par *Food and Drug Administration (FDA)* (www.cellexcovid.com). Pour les sujets symptomatiques, la détection de l'ARN viral était fait par le RT- PCR 4 canaux TL-988 (Toption Group Co. Ltd) et les examens hématologiques et biochimiques de routine ont été également réalisés.

**Définitions opérationnelles:** dans la présente étude, les sujets séropositifs au SARS-CoV-2 étaient ceux chez qui les anticorps IgG et/ou IgM anti-SARS-CoV-2 étaient détectés. La présence isolée des IgM indiquait une exposition récente au SARS-CoV-2 entre 3 et 6 jours et la présence des IgG indiquait une exposition prolongée supérieure à 8 jours [6].

**Analyses statistiques:** le logiciel MedCalc® version 18,11 a été utilisé pour les analyses statistiques. Les données sont présentées, selon le cas, par la moyenne (±DS) ou la fréquence relative en pourcent. Le test Khi2 a été utilisé pour la comparaison des variables qualitatives. La probabilité de la séroprévalence anti-SARS-CoV-2 en fonction des facteurs de risque supposés a été modélisée dans une régression logistique multiple. Une valeur de p < 0.05 définissait le seuil de signification statistique.

## Résultats

**Caractéristiques générales:** Tableau 1 montre les caractéristiques générales de la population étudiée. Au total, six cent quatre-vingt-quatre (684) sujets, voyageurs ou travailleurs, ont été dépistés entre mai et août 2020. La moyenne d´âge des sujets dépistés était de 41,7±14,5 ans. Respectivement 13,3% étaient âgés de 60 ans et plus, 63,7% étaient des hommes (p < 0,0001), 630 (92,1%) autochtones, 19 (2,8%) autres africains, 29 (4,2%) hispaniques et 6 (0,9%) asiatiques.

**Tableau 1:** caractéristiques générales des voyageurs et travailleurs dépistés entre mai et août 2020 à la clinique Saint Luc de Bukavu

Variables	Groupe entier	Séronégatifs	Séropositifs asymptomatiques	Séropositifs symptomatiques
**Genre**				
**Hommes et femmes, n(%)**	684(100)	405(59,2)	240(35,1)	39(5,7)
**Hommes, n(%)**	436(100)	259(59,4)	154(35,3)	23(5,3)
**Femmes, n(%)**	248(100)	146(58,9)	86(34,7)	16(6,5)
**p**	-	-	-	0.52
**Age(ans), moy±DS**	41,7±14,5	39,6±14,1	43,4±14,0*	53,5±15,0**
**<40, n(%)**	325(100)	217(66,8)	100(30,8)	8(2,5)
**40-59, n(%)**	268(100)	146(54,5)	107(39,9)	15(5,6)
**≥60, n(%)**	91(100)	42(46,2)	33(36,3)	16(17,6)
**p**	-	-	-	<0,0001
**Origine**				
**Congolaise, n(%)**	630(100)	377(59,8)	216(34,3)	37(5,9)
**Africaine autre, n(%)**	19(100)	12(63,2)	7(36,8)	0(0,0)
**Hispanique, n(%)**	29(100)	13(44,8)	15(51,7)	1(3,4)
**Asiatique, n(%)**	6(100)	3(50,0)	2(33,3)	1(16,7)
**p**	-	-	-	0,42
**Profession**				
**Médicale, n(%)**	87(100)	68(78,2)	18(20,7)	1(1,1)
**Non médicale, n(%)**	597(100)	337(56,4)	222(37,2)	38(6,4)
**p**	-	-	-	0,05
**Symptômes**				
**Fièvre, n(%)**	-	-	-	30(76,9)
**Asthénie physique, n(%)**	-	-	-	24(61,5)
**Toux, n(%)**	-	-	-	20(51,3)
**Céphalées, n(%)**	-	-	-	20(51,3)
**Dyspnée, n(%)**	-	-	-	15(38,5)
**Douleurs thoraciques, n(%)**	-	-	-	8(20,5)
**Troubles neurologiques, n(%)**	-	-	-	3(7,7)
**Diarrhées et/ou vomissements, n(%)**	-	-	-	1(2,6)

* p<0,05 comparé aux séronégatifs ; **p<0,05 comparé aux séropositifs asymptomatiques

**Figure 1:**
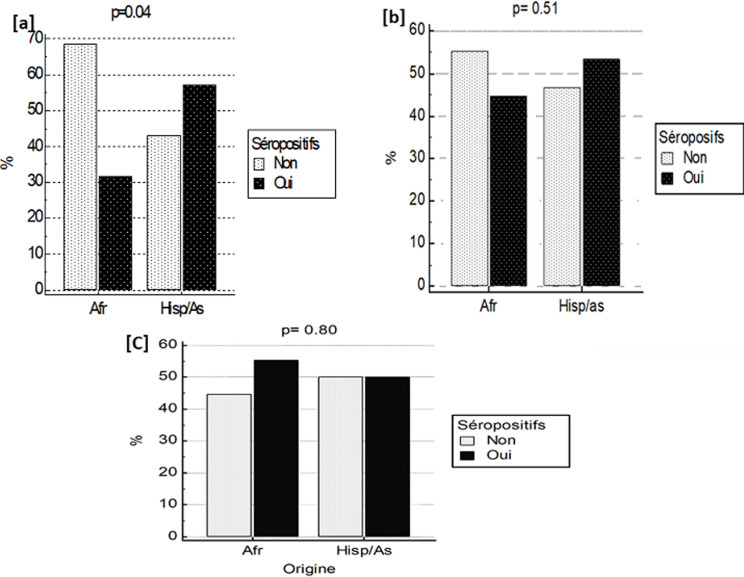
séroprévalence anti-SARS-CoV-2 en fonction de l´âge et d´origine; Afr: origine congolaise et autre africaine; Hisp: origine hispanique; As: origine asiatique; (A) chez des sujets de moins de 40 ans; (B) chez des sujets entre 40 et 59 ans; (C) chez des sujets de 60 ans et plus

**Figure 2:**
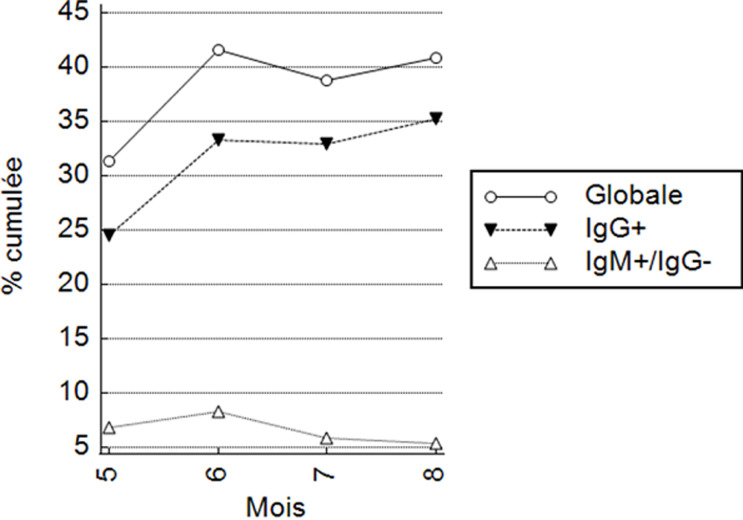
séroprévalence anti-SARS-CoV-2 cumulée globale, des IgG et des IgM

**Séroprévalence des anticorps anti-SARS-CoV-2:** la sérologie anti-SARS-CoV-2 est reprise dans le Tableau 2 et dans la Figure 1. Parmi les 684 sujets testés, 279 (40,8%) étaient séropositifs pour le SARS-CoV-2 parmi lesquels 238 (34,7%) avaient des IgG+/IgM+, 4(0,5%) des IgG+/IgM- et 37(5,4%) des IgG-/IgM+. Comparés aux sujets < 40 ans, les sujets entre 40 et 59 ans et ceux ≥ 60 ans avaient respectivement 1,67 fois et 2,34 fois plus fréquemment des Ac anti-SARS-CoV-2 (p < 0,05). Les non professionnels de santé avaient 2,76 fois plus fréquemment les Ac anti-SARS-CoV-2 comparé au personnel médical (p < 0,01). Enfin, la séropositivité anti-SARS-CoV-2 était plus fréquente chez les hispaniques/Asiatiques que chez les congolais/autres africains (OR brut = 1,77; p=0,09), au mois de juin (OR brut = 2,07; p = 0,006) et au mois d´août (OR brut = 1,73; p = 0,02) comparé au mois de mai. Cependant, dans l´analyse multivariée, les prédicteurs indépendants de la séropositivité anti-SARS-CoV-2 était surtout l´âge > 60 ans (OR ajusté = 2,07; p = 0,003) et la non appartenance au corps médical (OR ajusté = 2,28; p = 0,009). La Figure 2 montre la séroprévalence cumulée des anticorps anti-SARS-CoV-2. La séroprévalence cumulée des IgG anti-SARS-CoV-2 est passée de 24,5% au mois de mai à 35,3% au mois d´août. La séroprévalence cumulée des IgM anti-SARS-CoV-2 est passée de 6,6% au mois de mai à 8,3% au mois de juin pour s´infléchir à 5,4% au mois d´août.

**Tableau 2:** séroprévalence anti-SARS-CoV-2 chez les voyageurs et travailleurs dépistés entre mai et août 2020

	Nombre	Séropositifs n(%)	Séronégatifs n(%)	OR brut (IC à 95%)	OR ajusté (IC à 95%)
**n(%)**	684(100)	279(40,8)	405(59,2)	-	-
**Age (ans)**					
**<40**	325(47,4)	108(33,2)	217(66,8)	1	1
**40-59**	268(39,2)	122(45,5)	146(54,5)	1,67(1,20-2,34)*	1,56(1,10-2,20)
**≥60**	91(13,3)	49(53,8)	42(46,2)	2,34(1,46-3,75)*	2,07(1,26-3,38)
**Sexe**					
**Femmes**	248(36,3)	102(41,1)	146(58,9)	1	-
**Hommes**	436(63,7)	177(40,6)	259(59,4)	0,94(0,65-1,34)	-
**Origine**					
**Africaine**	649(94,9)	260(40,1)	389(59,9)	1	1
**Non africaine**	35(5,1)	19(54,3)	16(45,7)	1,77(0,89-3,51)*	1,61(0,79-3,25)
**Personnel médical**					
**Oui**	87(12,7)	19(21,8)	68(78,2)	1	1
**Non**	597(87,3)	260(43,6)	337(56,4)	2,76(1,61-4,70)*	2,28(1,22-4,26)
**Mois**					
**Mai**	102(14,9)	32(31,4)	70(68,6)	1	1
**Juin**	150(21,9)	73(48,7)	77(51,3)	2,07(1,22-3,51)*	1,43(0,79-2,57)
**Juillet**	188(27,5)	66(35,1)	122(64,9)	1,18(0,70-1,97)	0,78(0,43-1,42)
**Aout**	244(35,7)	108(44,3)	136(55,7)	1,73(1,06-2,83)*	1,18(0,66-2,11)

**Présentation clinique des sujets symptomatiques:** parmi les 277 séropositifs pour le SARS-CoV-2, 39 (13,9%) étaient symptomatiques et hospitalisés. La moyenne d´âge était de 54,0±14,9 ans. Les symptômes les plus fréquents étaient la fièvre (76,9%), l´asthénie physique (61,5%), la toux (51,3%) et les céphalées (51,3%).

## Discussion

La présente étude note que, parmi 684 habitants de la ville de Bukavu dépistés de la COVID-19, 40,5% étaient séropositifs. Les prédicteurs indépendants des anticorps anti-SARS-CoV-2 étaient surtout l´âge > 60 ans et le fait de ne pas appartenir au personnel médical. Quatorze pourcent des séropositifs pour les SARS-CoV-2 étaient symptomatiques et hospitalisés. La présente étude est la première à avoir analysé la séroprévalence anti-SARS-CoV-2 en RDC. Bien que le dépistage ait été réalisé en milieu hospitalier, le motif de celui-ci était plus professionnel ou lié au voyage. Ainsi, ces résultats donnent un aperçu général de la communauté dans cette ville de l´Est de la RDC.

Trois observations majeures sont à relever. Premièrement, la séroprévalence anti-SARS-CoV-2 est très élevée (40,5%) et a évolué très rapidement en trois mois. Cette séroprévalence est significativement plus élevée que celle relevée par des données des études aux Etats-Unis qui était de 13,7% parmi le personnel médical [8] et 4,0% dans la population adulte de Los Angeles [9], en Chine qui était de 2,1% parmi les visiteurs dans les hôpitaux [10], en Allemagne qui était de 0,9% parmi les donneurs du sang [11] ou en Corée qui était de 7,6% chez des patients et les gardes malades sans histoire de la COVID-19 [12]. La séroprévalence des IgG trouvée dans notre étude était également significativement plus élevée que celle relevée à Chelsea dans la population générale qui était de 22,5%. Ces résultats témoignent donc d´un contact massif de la population étudiée avec le SARS-CoV-2. Il est à noter que la différence de contamination n´était pas significative entre les autochtones et non autochtones (autres africains, asiatiques ou hispaniques) suggérant le rôle important des facteurs environnementaux. Et comme énoncé ci-haut, ces résultats reflètent probablement la situation dans la population générale car les sujets dépistés étaient pour la plupart des voyageurs ou travailleurs en quête d´un certificat médical. La raison majeure pouvant expliquer cette séroprévalence élevée est l´absence d´application des mesures de prévention par la population générale quel qu´en soit l´origine notamment le confinement strict, la distanciation sociale et le port des masques. Ces mesures n´ont jamais été respectées pour des raisons économiques et sociologiques. Dans la présente étude, les sujets n´appartenant pas au personnel médical étaient plus affectés. Certainement, le personnel médical était plus conscient de l´existence de la maladie que la population générale. De même, la tranche d´âge la plus affectée était celle de 60 ans et plus, certainement plus vulnérable du fait d´une expression plus accrue de l´enzyme de conversion d´angiotensine 2 par les cellules cibles dans cette tranche d´âge comparée aux tranches d´âges plus jeunes [13].

La deuxième observation est la diminution significative de la séroprévalence des IgM+/IgG- entre juin et août dénotant une diminution des sujets ayant été exposés récemment au SARS-CoV-2. Cette évolution de la séroprévalence parmi les voyageurs et travailleurs dans notre étude était similaire à l´évolution épidémiologique de la COVID-19 dans la population générale tel que publié par le comité national de riposte contre la COVID-19 qui montrait une diminution des nouveaux cas à partir du mois de juillet 2020 parmi les sujets dépistés par RT-PCR [14].

Cependant, la séroprévalence > 40% relevée dans notre étude était significativement plus élevée que la proportion des cas positifs dépistés par le RT-PCR dans la population générale [14]. Nos résultats corroborent ainsi ceux d´autres auteurs qui ont démontré que le taux d´infection relevée par la séroprévalence anti-SARS-CoV-2 serait 30 à 50 fois plus élevé que celui détecté par le PCR [15]. Et il est certain que le nombre de personnes probablement immunisées soit sous-estimé du fait que nous ne pouvions pas évaluer l´immunité cellulaire dans la présente étude. Ainsi, Il est possible, qu´avec une séroprévalence des anticorps anti-SARS-CoV-2 de 40,8%, une immunité collective se soit développée dans la population générale.

Enfin, dans la présente étude, seuls 14% étaient symptomatiques vers 86% asymptomatiques. Les symptômes évoqués sont ceux relevés dans d´autres communautés notamment en Chine [16] et en Occident [17]. Nos observations corroborent ainsi la littérature. En effet, la COVID-19 est en réalité une maladie bénigne mais s´aggravant chez certains sujets à risque. Le présent travail doit être interprété en tenant compte de ses limites. Premièrement, le dépistage s´est réalisé en milieu hospitalier, ce qui pourrait expliquer un biais de sélection. Mais cette limite est à minimiser parce que la plupart des sujets dépistés étaient des voyageurs ou des travailleurs en quête d´un certificat médical. Deuxièmement, l´utilisation des tests sérologiques rapides est une limite majeure dans la présente étude en ce sens que certains individus pourraient avoir des niveaux d´anticorps indétectables ou être séronégatifs malgré une exposition au SARS-CoV-2. En effet, la réponse humorale ne constitue pas la seule preuve d´immunité contre la COVID-19. Ainsi, nos données épidémiologiques reposant uniquement sur la détection des anticorps dirigés contre le SARS-CoV-2 pourraient conduire à une sous-estimation de l'exposition antérieure au virus. Enfin, nous n´avons pas utilisé la technique immuno-enzymatique (Elisa) qui est plus performante et qui aurait permis de quantifier les anticorps du sujet présents dans le sang.

## Conclusion

La présente étude montre une séroprévalence très élevée des anticorps anti-SARS-CoV-2 parmi les voyageurs et travailleurs dépistés entre mai et août 2020 dans la ville de Bukavu. Ces résultats suggèrent une exposition massive de ce groupe étudié et, par extension, de la population générale au SARS-CoV-2 pouvant impacter positivement sur l´immunité communautaire dans cette région. Ainsi, la prise en charge de la COVID-19 devrait être contextualisée en fonction des réalités de chaque région.

### Etat des connaissances sur le sujet

- Le nombre des cas confirmés par RT PCR et des décès parmi ceux-ci reste faible en Afrique subsaharienne comparées à d´autres régions du monde;
- Le nombre des cas de COVID-19 diminue depuis le mois de juillet 2020 en Afrique subsaharienne.

### Contribution de notre étude à la connaissance

- La séroprévalence anti-SARS-CoV-2 est très élevée parmi les voyageurs et travailleurs dans la ville de Bukavu suggérant une exposition massive de la population au SARS-CoV-2, ce qui pourrait impacter positivement sur l´immunité collective et, ainsi, le profil épidémiologique de la COVID-19.

## Remerciements

Notre gratitude au Ministère Provincial de la Santé et à la Division Provinciale de la Santé du Sud-Kivu pour avoir sélectionné la clinique Saint Luc de Bukavu parmi les centres de dépistage de la COVID-19 pour les voyages nationaux. Ce qui nous a permis de constituer un échantillon pour la présente étude.
